# Supplementary material for: Phytochemical Screening and Antioxidant and Cytotoxic Effects of Acacia macrostachya
Source: Plants (Basel). 2021 Jul 2;10(7):1353. doi: 10.3390/plants10071353 (PMC8309326; doi:10.3390/plants10071353)
Supplement: Supplementary file 1 [file plants-10-01353-s001.zip › plants-1245755-supplementary.pdf]

plants

Supplementary documents

Phytochemical Screening and Antioxidant and Cytotoxic Effects of *Acacia macrostachya*

Hamidou Têeda Ganamé [1,2], Yssouf Karanga [1,3], Issa Tapsoba [1], Mario Dicato [2], Marc F. Diederich [4], Claudia Cerella [2,†,*] and Richard Wamtinga Sawadogo [5,†,*]

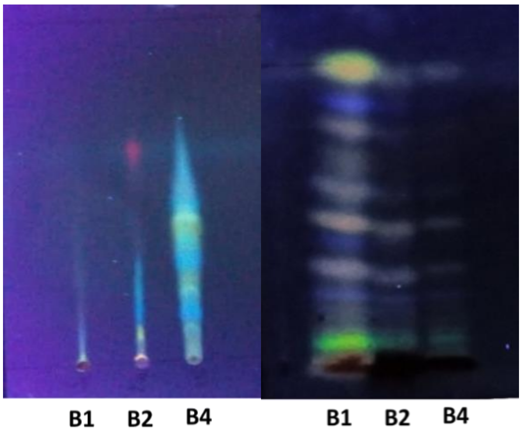

(**a**) (**b**)

Figure S1. Qualitative phytochemical analysis by TLC. (**a**): Flavonoid; Mobile phase: Chloroform-Ethyl acetate (60/40 *v/v*); (**b**): Terpenoids/Steroids; Mobile phase: Hexane-Ethyl acetate (20/4 *v/v*). In the first plaque (**a**) submitted to UV light at 365 nm, the spots of flavonoids appear yellow, green, orange, or fluorescent blue. The second plate (**b**) revealed with the Liebermann-Burchard reagent characterize the presence of terpenoids and/or steroids in green and purple.

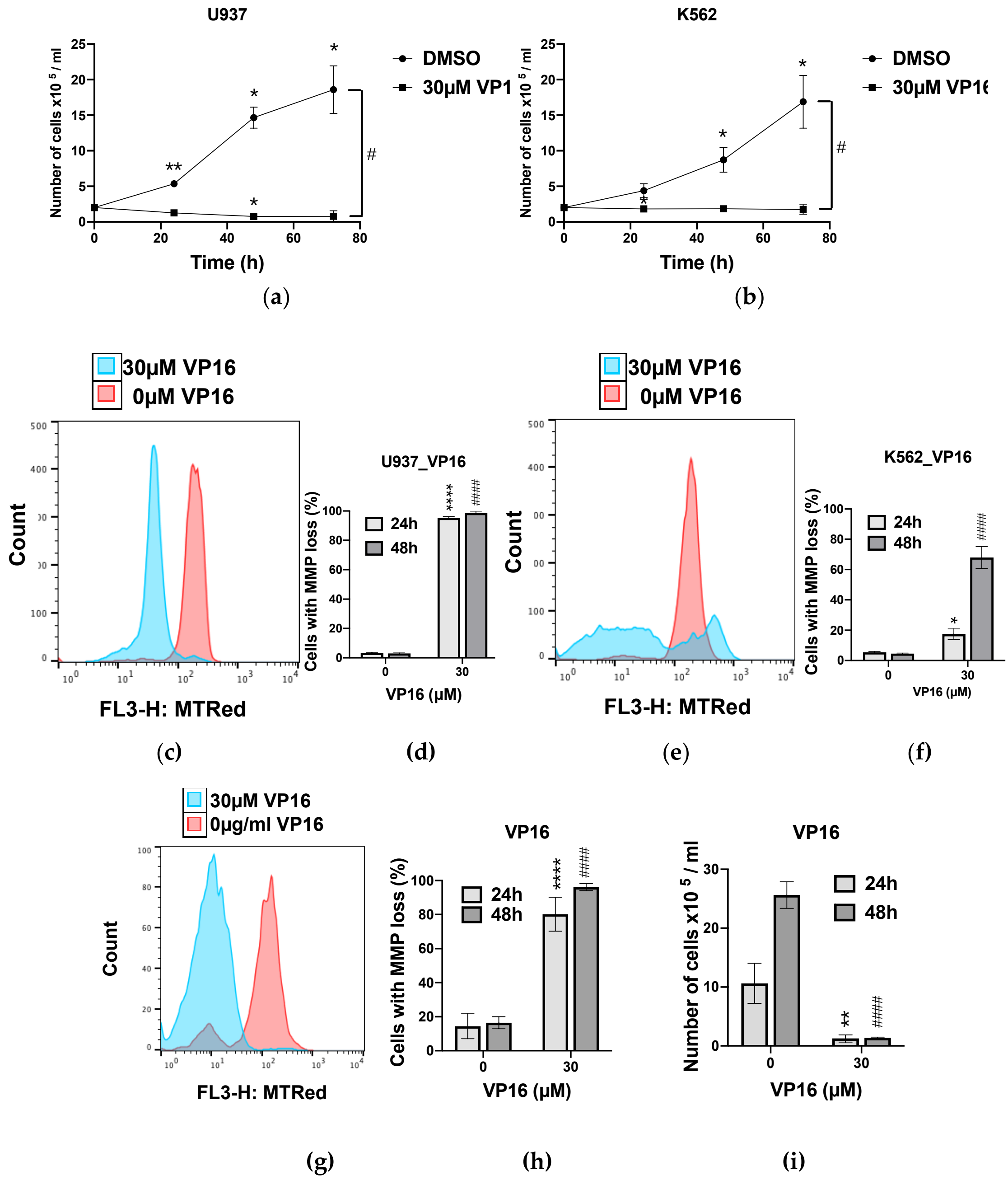

Figure S2. Effects of the positive control etoposide (VP16) on U937 (**a, c, d**), K562 (**b, e, f**) cells and proliferating PBMCs (**g, h, i**) by Hoechst and Mito Tracker® Red staining analysis and trypan bleu assay after 24h and 48h treatment; ANOVA Two-way; Post-hoc: Sidak *,#: p < 0.05; **: p< 0.01; ****, ####: p < 0.0001.

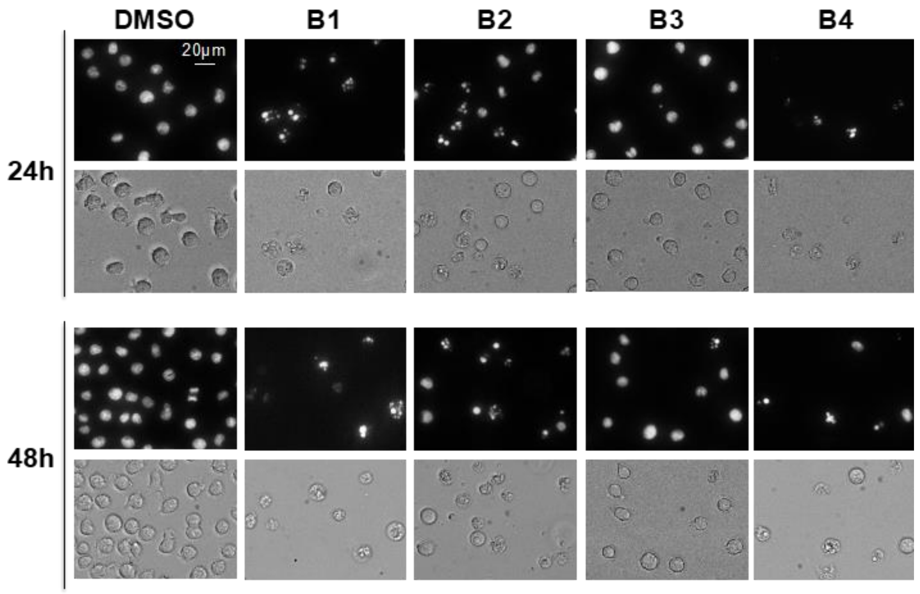

(a)

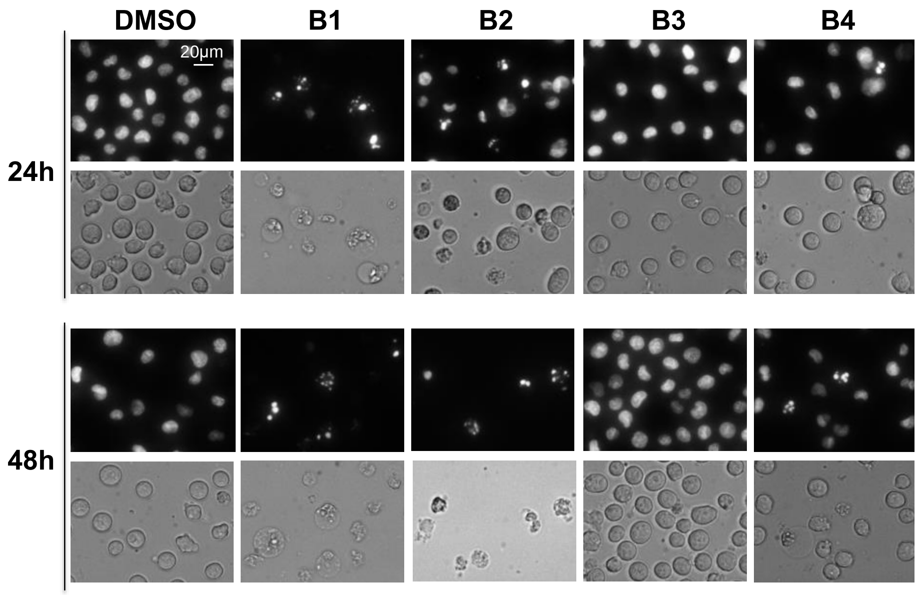

(b)

Figure S3. Apoptotic nuclear fragmentation induced by different extracts at 50 μg/mL in U937 (**a**) and K562 (**b**) cells was analyzed after Hoechst staining (one representative of three independent experiments).
